# Increased Incidence of Early-Onset Colorectal Cancer in Low Sociodemographic Index Countries: Higher Rising Burden in Young Females

**DOI:** 10.7759/cureus.38998

**Published:** 2023-05-14

**Authors:** Pojsakorn Danpanichkul, Sorawit Ongsupankul, Pinyada Moolkaew, Ranchana Yamsiri, Nuttada Panpradist

**Affiliations:** 1 Immunology Unit, Department of Microbiology, Chiang Mai University, Chiang Mai, THA; 2 Department of Internal Medicine, John A. Burns School of Medicine, University of Hawaiʻi, Honolulu, USA; 3 Immunology Unit, Department of Microbiology, Faculty of Medicine, Chiang Mai University, Chiang Mai, THA; 4 Global Center for Integrated Health of Women, Adolescents, and Children (Global WACh), University of Washington, Seattle, USA

**Keywords:** low sociodemographic index, female preponderance, screening, gender disparities, colorectal cancer, young-onset colon cancer, sporadic early-onset colorectal cancer

## Abstract

Introduction

Early-onset colorectal cancer (EOCRC) is becoming a growing concern due to its increased incidence among younger individuals, particularly in areas with limited healthcare access and funding, such as in countries with a low sociodemographic index (SDI). However, there are limited studies regarding this problem. Therefore, our study primarily aims to address the dearth of knowledge in this area by assessing the trends in EOCRC in low SDI countries over 10 years.

Methods

In this study, we analyzed data from the Global Burden of Disease Study 2019 to investigate the changes in EOCRC over time in low SDI countries. Our analysis involved determining the yearly frequencies and age-standardized rates (ASRs) of EOCRC incidence, death, and disability-adjusted life years (DALYs) by gender.

Results

In 2019, the number of newly diagnosed EOCRC cases in low SDI countries was 7,716, while the global cases were 225,736. The incidence rates of EOCRC increased significantly higher in low SDI countries compared to the global average between 2010 and 2019, with a 1.38-fold higher increase among females. Mortality rates and DALYs also increased in the low SDI countries, with the annual percentage change from 2010 to 2019 of 0.96 (95% uncertainty interval (UI): 0.88-1.03) and 0.91 (95% UI: 0.83-0.98), respectively.

Conclusion

Our research highlights a significant rise in CRC in low SDI countries, particularly in the female population. Therefore, it emphasizes the need for prompt and efficient interventions, including but not limited to effective screening methods and mitigation of risk factors.

## Introduction

Colorectal cancer (CRC) is a leading cause of preventable morbidity and mortality worldwide. It is the third most diagnosed cancer [1]. However, individuals from low socioeconomic backgrounds and certain racial minorities are disproportionately affected by CRC, which may be attributed to disparities in incidence and outcomes [2]. These disparities may stem from differences in exposure to risk factors, such as unhealthy diet and sedentary lifestyle, limited access to risk-reducing interventions, such as screening and follow-up of abnormal test results, and lack of access to high-quality treatment resources. These factors operate at multiple levels, including the individual, provider, health system, community, and policy, which likely account for these CRC epidemiological disparities [3].

Interestingly, recent epidemiological trends have highlighted a concerning rise in early-onset colorectal cancer (EOCRC), referring to cancers diagnosed before the age of 50 years, linked to exposure to multiple risk factors during early life, including obesity and diabetes [4,5]. It is noteworthy that EOCRC displays more indolent clinical features and poorer overall survival rates [6].

According to our PubMed search using the search terms “incidence” OR “screening” AND “early-onset colorectal cancer” OR “young-onset colorectal cancer” AND “low SDI countries” from conception to February 2023, there is a lack of comprehensive research on EOCRC in low sociodemographic index (SDI) countries, which exhibits multiple barriers to health care, including but not limited to insufficient healthcare infrastructure and lack of routine screening [7]. Our study aims to address this critical knowledge gap by assessing the temporal trends of this disease in these regions, shedding light on the unique challenges these populations face.

## Materials and methods

Data source

This study drew upon data from the Global Burden of Disease Study 2019 (GBD 2019) [8]. The GBD 2019 is a global effort to evaluate the prevalence of diseases and associated risk factors in 204 countries and territories. This study examined the incidence, mortality, and disability-adjusted life years (DALYs) related to EOCRC across 47 low SDI countries. The data for this analysis were obtained from the Global Health Data Exchange (GHDx) query tool, a curated online catalog of health-related data maintained by the Institute for Health Metrics and Evaluation.

Study aims and definitions

EOCRC was defined as CRC diagnosed in patients aged 15-49 years, and International Classification of Diseases, Tenth Revision (ICD-10) codes C18, C19, and C20 were used to map the presence of EOCRC [9]. The main objective of this study is to examine the occurrence of EOCRC in low SDI countries, including Afghanistan, Angola, Benin, Burkina Faso, Burundi, Central African Republic, Chad, Comoros, the Democratic Republic of the Congo, Djibouti, Equatorial Guinea, Eritrea, Ethiopia, Gambia, Guinea, Guinea-Bissau, Haiti, Kiribati, Lesotho, Liberia, Madagascar, Malawi, Mali, Mauritania, Mozambique, Myanmar, Niger, Nigeria, Papua New Guinea, the Republic of the Congo, Rwanda, Sao Tome and Principe, Senegal, Sierra Leone, Solomon Islands, Somalia, South Sudan, Sudan, Swaziland, Tanzania, Togo, Uganda, Vanuatu, Yemen, Zambia, and Zimbabwe. The full methodology used to estimate the disease burden of CRC in GBD 2019 has been previously described [10]. In this study, data on EOCRC were collected from various sources, such as population-based cancer registries, vital registration systems, or verbal autopsy studies. The data quality from each country/territory was assessed and rated on a scale ranging from 0 to 5, with 5 representing the highest quality. To reduce data heterogeneity, misclassification correction, garbage code redistribution, and noise reduction algorithms were employed. Mortality-to-incidence ratios were used to estimate CRC and subgroup analysis was conducted to assess gender differences. The incidence rate was calculated by dividing the number of newly diagnosed cases of CRC during a defined period by the population size at risk during the same period. The study employed the Cause of Death Ensemble model, a Bayesian geospatial regression analysis, to estimate CRC-related mortality by age, gender, country/region, and year.

Data and statistical analysis

The direct method was used to calculate age-standardized rates (ASRs) based on the GBD 2019 population estimate with five-year age groups [8]. These estimates and 95% uncertainty intervals (UIs) were reported, representing the values between the 2.5th and 97.5th percentiles across 1,000 draws from a posterior distribution. Age-specific incidence rates (ASIRs) are calculated by dividing the number of new cases in a specific age group by the total population in that age group, then multiplying by 100,000. Age-specific death rates (ASDRs) are determined by dividing the number of deaths in a specific age group by the population in that age group and multiplying by 100,000. DALYs combine years lost and years lived with disability. Years of life lost are calculated by subtracting the age at death from the standard life expectancy. In contrast, years lived with disability are found by multiplying the number of individuals with a specific health condition by the average duration and a disability weight reflecting the condition severity. Age-standardized DALY rates are obtained by applying age-specific DALY rates to a standard population. To calculate the percentage change between 2010 and 2019 for any category, the value difference from the two years is divided by the value in 2010. The Joinpoint Regression Program version 4.6.1.0 (Statistical Research and Applications Branch, National Cancer Institute, Bethesda, Maryland) was used to estimate the annual percentage change (APC) and corresponding 95% UIs for temporal changes in ASRs between 2010 and 2019. An increasing trend is identified when both the APC and the lower boundary of the 95% UI are positive, while a decreasing trend is identified when both the APC and the upper limit of the 95% UI are negative.

## Results

Incidence of EOCRC worldwide and low SDI countries

**Figure 1:**
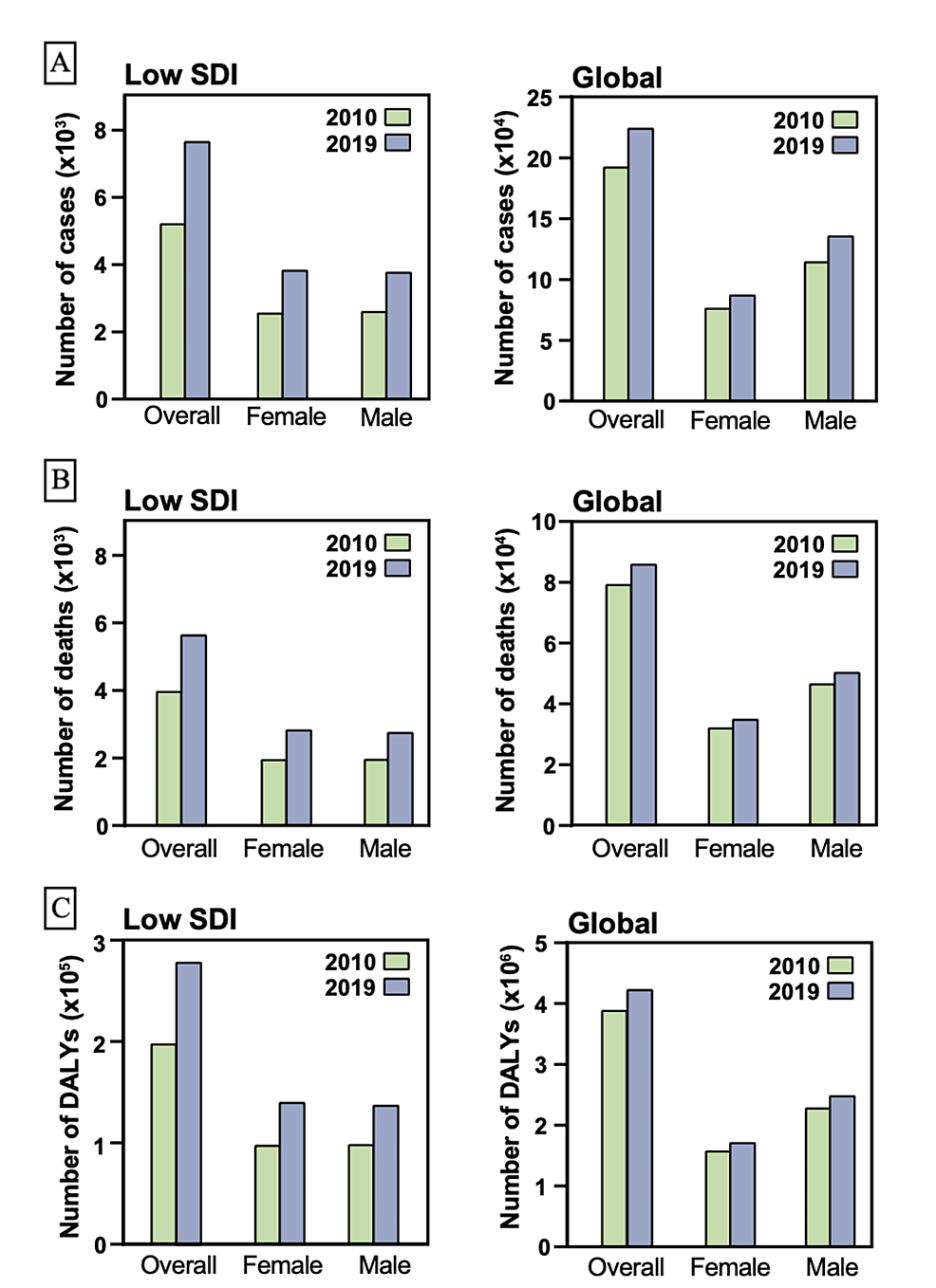
(A) Number of cases diagnosed with CRC in individuals aged 15-49 years in 2010 and 2019. (B) Number of deaths in patients with CRC aged 15-49 years in 2010 and 2019. (C) Number of DALYs in patients with CRC aged 15-49 years in 2010 and 2019. CRC: colorectal cancer; DALYs: disability-adjusted life years; SDI: sociodemographic index.

Globally, there were 225,736 newly diagnosed cases of EOCRC, with 7,716 of these cases being diagnosed in countries with a low SDI in 2019. The incidence rates of CRC in patients aged 15-49 years increased significantly globally and in low SDI countries (Figure 1). Specifically, the ASIR of CRC exhibited an APC of 1.33 (95% UI: 1.23 to 1.43, Table 1) in low SDI countries, higher than the global APC of 0.95 (95% UI: 0.8 to 1.1). The overall incidence rate of CRC in low SDI countries increased by 26.24% from 8.88 (95% UI: 8.21 to 9.7) to 11.21 (95% UI: 9.68 to 12.92) per 100,000, with a more significant increase observed in females than males with APC of 1.54 (95% UI: 1.41 to 1.66) and 1.12 (95% UI: 0.96 to 1.28), respectively. Despite the difference in the degree of increase, ASIRs in 2019 in low SDI countries in both genders were comparable in both genders. Global APC of ASIR, however, underwent a higher change in males compared to females with APC of 1.13 (95% UI: 0.87 to 1.4) and 0.66 (95% UI: 0.48 to 0.84), respectively. Accordingly, ASIRs in 2019 were higher in males compared to females with ASIR of 6.89 (95% UI: 6.17 to 7.75) and 4.55 (95% UI: 4.11 to 5.02) per 100,000, respectively.

**Table 1:** Age-specific incidence rates of colorectal cancer in patients aged 15-49 years in 2010 and 2019. APC: annual percentage change; ASIR: age-specific incidence rate; SDI: sociodemographic index; 95% UI: 95% uncertainty interval.

Incidence	2010 incidence (95% UI)	2010 ASIR per 100,000 (95% UI)	2019 incidence (95% UI)	2019 ASIR per 100,000 (95% UI)	APC (95% UI)	p
Low SDI						
Overall	5,267.29 (4,666.97 to 5,971.02)	1.27 (1.13 to 1.44)	7,716.42 (6,642.97 to 8,911.86)	1.43 (1.23 to 1.65)	1.33 (1.23 to 1.43)	<0.001
Female	2,611.14 (2,278.98 to 2,962.44)	1.25 (1.09 to 1.42)	3,886.86 (3,304.13 to 4,557.89)	1.43 (1.21 to 1.67)	1.54 (1.41 to 1.66)	<0.001
Male	2,656.14 (2,310.81 to 3,175.89)	1.29 (1.12 to 1.54)	3,829.56 (3,228.24 to 4,555.04)	1.43 (1.2 to 1.7)	1.12 (0.96 to 1.28)	<0.001
Global						
Overall	193,852.68 (185,696.51 to 204,819.33)	5.27 (5.04 to 5.56)	225,736.01 (207,658.04 to 246,755.67)	5.74 (5.28 to 6.27)	0.95 (0.8 to 1.1)	<0.001
Female	77,898.53 (74,083.38 to 81,580.87)	4.27 (4.06 to 4.47)	88,597.7 (79,973.86 to 97,559.69)	4.55 (4.11 to 5.02)	0.66 (0.48 to 0.84)	<0.001
Male	115,954.15 (10,9552.76 to 125,436.89)	6.24 (5.89 to 6.75)	137,138.3 (122,715.32 to 154,229.08)	6.89 (6.17 to 7.75)	1.13 (0.87 to 1.4)	<0.001

Mortality and DALYs related to EOCRC worldwide and low SDI countries

**Figure 2:**
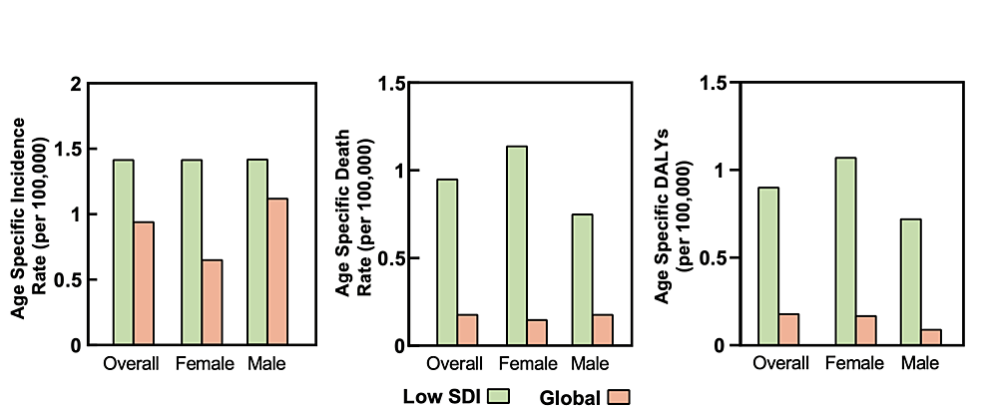
Age-specific rates of patients diagnosed with CRC aged 15-49 years in low SDI countries and globally. CRC: colorectal cancer; DALYs: disability-adjusted life year; SDI: sociodemographic index.

The mortality and disability rates for EOCRC were increasing at a higher rate in low SDI countries compared to the global average, as depicted in Figure 2. In terms of mortality, there were 86,545 deaths attributed to EOCRC globally, and 5,693 of these deaths occurred in low SDI countries (Table 2). The incremental change in mortality rate was higher in low SDI countries compared to the global rate, with APC of 0.96 (95% UI: 0.88 to 1.03, Table 2) and 0.18 (95% UI: 0.07 to 0.29), respectively. When stratified by gender, the degree of change in low SDI countries was higher in males compared to females, with APC of 1.15 (95% UI: 1.03 to 1.28) and 0.76 (95% UI: 0.62 to 0.9), respectively. However, similar to the ASIRs, the ASDRs in 2019 were comparable in both genders.

**Table 2:** Age-specific death rates of colorectal cancer in patients aged 15-49 years in 2010 and 2019. APC: annual percentage change; ASDR: age-specific death rate; SDI: sociodemographic index; 95% UI: 95% uncertainty interval.

Mortality	2010 deaths (95% UI)	2010 ASDR per 100,000 (95% UI)	2019 deaths (95% UI)	2019 ASDR per 100,000 (95% UI)	APC (95% UI)	p
Low SDI						
Overall	4,025.23 (3,551.64 to 4,553.55)	0.97 (0.86 to 1.1)	5,693.01 (4,932.99 to 6,582.54)	1.05 (0.91 to 1.22)	0.96 (0.88 to 1.03)	<0.001
Female	2,007.91 (1,735.18 to 2,270.07)	0.96 (0.83 to 1.09)	2,882.46 (2,457.62 to 3,366.93)	1.06 (0.9 to 1.24)	1.15 (1.03 to 1.28)	<0.001
Male	2,017.32 (1,744.54 to 2,409.56)	0.98 (0.85 to 1.17)	2,810.56 (2,384.43 to 3,405.87)	1.05 (0.89 to 1.27)	0.76 (0.62 to 0.9)	<0.001
Global						
Overall	79,880.91 (76,380.48 to 84,491.54)	2.17 (2.07 to 2.29)	86,545.55 (80,161.98 to 93,431.12)	2.2 (2.04 to 2.37)	0.18 (0.07 to 0.29)	0.002
Female	32,705.47 (30,922.16 to 34,597.37)	1.79 (1.7 to 1.9)	35,545.94 (32,351.47 to 38,886.07)	1.83 (1.66 to 2)	0.15 (0.01 to 0.28)	0.03
Male	47,175.44 (44,734.51 to 50,460.37)	2.54 (2.41 to 2.71)	50,999.61 (45,982.79 to 56,178.55)	2.56 (2.31 to 2.82)	0.18 (0.12 to 0.24)	<0.001

Regarding disability, age-specific DALYs (ASDALYs) were higher in the global population compared to low SDI countries with ASDALYs of 108.25 (95% UI: 100.2 to 116.67; Table 3 and Figure 1C) and 51.95 (95% UI: 44.96 to 60.01), respectively. The APC of ASDALYs was 0.91 (95% UI: 0.83 to 0.98) in low SDI countries, which was five times higher than the change in the global average, with an APC of 0.16 (95% UI: 0.09 to 0.22). The increase in low SDI countries’ DALYs was mainly attributed to females, with an APC of 1.08 (95% UI: 0.97 to 1.2) compared to the APC of 0.73 (95% UI: 0.59 to 0.86) in males. However, DALYs were stable in global females (p = 0.218).

**Table 3:** Age-specific disability-adjusted life years of colorectal cancer in patients aged 15-49 years in 2010 and 2019. APC: annual percentage change; ASDALYs: age-specific DALYs; DALYs: disability-adjusted life years; SDI: sociodemographic index; 95% UI: 95% uncertainty interval.

Disability	2010 DALYs (95% UI)	2010 ASDALYs per 100,000 (95% UI)	2019 DALYs (95% UI)	2019 ASDALYs per 100,000 (95% UI)	APC (95% UI)	p
Low SDI						
Overall	199,327.14 (175,652.8 to 225,558.04)	48.08 (42.37 to 54.41)	280,702.7 (242,945.92 to 324,265.95)	51.95 (44.96 to 60.01)	0.91 (0.83 to 0.98)	<0.001
Female	99,312.22 (86,059.67 to 112,109.92)	47.7 (41.34 to 53.85)	141,788.9 (120,702.07 to 165,972.82)	52.1 (44.35 to 60.98)	1.08 (0.97 to 1.2)	<0.001
Male	100,014.92 (86,464.21 to 119,354.5)	48.46 (41.9 to 57.83)	138,913.8 (117,617.21 to 168,361.1)	51.8 (43.86 to 62.78)	0.73 (0.59 to 0.86)	<0.001
Global						
Overall	3,917,689.56 (3,748,336.16 to 4,146,068)	106.41 (101.81 to 112.61)	4,259,922.01 (3,942,849.93 to 4,590,979.19)	108.25 (100.2 to 116.67)	0.16 (0.09 to 0.22)	<0.001
Female	1,604,997.1 (1,522,276.98 to 1,694,413.97)	88.03 (83.49 to 92.94)	1,744,761.22 (1,587,817.76 to 1,910,865.4)	89.69 (81.62 to 98.23)	0.15 (0.03 to 0.27)	0.014
Male	2,312,692.46 (2,195,533.05 to 2,476,103.8)	124.43 (118.13 to 133.22)	2,515,160.78 (2,270,968.53 to 2,755,869.19)	126.41 (114.13 to 138.5)	0.08 (-0.05 to 0.21)	0.218

## Discussion

The increasing incidence of EOCRC is a growing concern worldwide, as younger patients with CRC often present with more advanced diseases and have worse outcomes, including higher mortality rates, than older patients [4]. The underlying pathogenesis of EOCRC remains unclear; however, risk factors such as ethnicity, family history of CRC, and male sex have been identified. Moreover, it has been established that individuals with lower socioeconomic status are at a higher risk of CRC burden, including higher case fatality rate and late presentation stage [11,12]. Thus, this study aims to investigate the mounting burden of EOCRC in low SDI countries.

Our research revealed that individuals residing in low SDI countries bear a more significant increasing burden of EOCRC. Although the male sex is a known risk factor in traditional CRC and EOCRC, our study found that females had a higher burden of the disease and incremental burden changes than males [13-15]. This may be explained by a distinct set of challenges contributing to their elevated risk, including inadequate access to healthcare services, such as screening methods, and cultural and social barriers hindering their ability to obtain care [16]. Additionally, since screening for CRC in young adults is not routine, the reported incidence may underestimate the true prevalence.

This study has several strengths, notably the use of the most up-to-date and comprehensive Global Burden of Disease (GBD) estimate, which provides cause-specific rates categorized by age, gender, and location. However, it is crucial to recognize certain limitations of the study. The accuracy of the GBD estimate relies on the quality and inclusiveness of vital registration systems in each country, which can vary. In countries without reliable data, GBD estimate relies heavily on modeling techniques, predictive covariates, historical trends, or trends extrapolated from neighboring countries, leading to potential uncertainties in estimating the true burden of EOCRC. Another limitation is the low incidence of EOCRC, which can make diagnosis challenging and result in an underestimation of the actual burden of the disease. Nonetheless, this database provides valuable evidence of the increasing burden of this relatively rare disease, which is a matter of concern and required immediate action. Fortunately, numerous pieces of evidence demonstrate that early colon cancer screening has been a cost-effective intervention compared to no screening [17,18]. In addition, various methods can be utilized to reduce the burden, such as increasing public awareness campaigns to promote healthy lifestyles, implementing high-risk screening programs, and improving access to medical care. The present study highlights the need for early screening for CRC in low SDI countries, especially given the higher incidence of EOCRC in women. In addition, it is essential to take multiple measures to tackle the epidemiological disparities and alleviate the burden of EOCRC. However, before implementing such measures, it is important to perform cost-effectiveness analyses and identify at-risk groups, which would enhance the efficacy of these interventions. Overall, this study emphasizes the importance of prioritizing EOCRC as a public health concern in these countries and subsequently developing comprehensive approaches to address this growing problem.

## Conclusions

The findings of our study uncovered a noteworthy increase in the prevalence of EOCRC in low SDI countries, with a specific emphasis on the female population. This revealed a pressing need for rapid and efficient interventions. Hence, a comprehensive strategy encompassing risk management measures, improved disease awareness, and effective screening techniques should be expeditiously implemented to address the surging burden of EOCRC.
